# 

**Published:** 1891-07-25

**Authors:** 


WITH EXTRA NURSING SUPPLEMENT.

Per Annum, 6s. 6d.; post free.

Monthly Parts, 6d.; post free 8d.

Ditto per Annum, 8s., post free.

### THE HOSPITAL

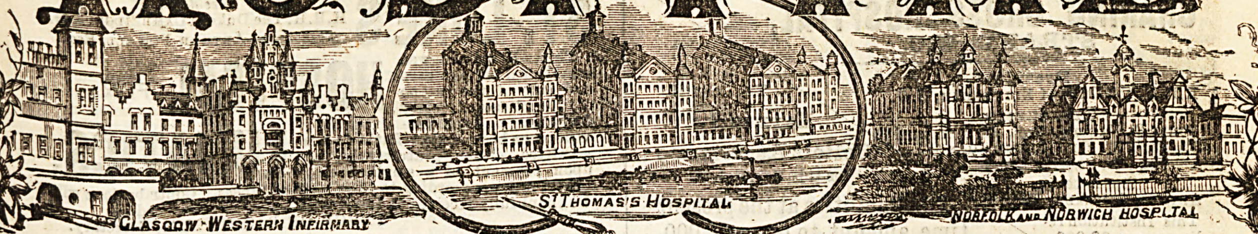

A WEEKLY JOURNAL OF

Science, Medicine, Nursing and Philanthropy.

### SATURDAY, JULY 25th, 1891.

**The National Liver** . . . 193
**Passing Topics.**—"Continentalising" the Sunday . . 194
**The Doctor's Armchair.**—Preparing for Flight . . 195
**Employments for Women.**—II. Type-writing for Ladies 196
**The Princess of Wales' Fund for Mrs. Grimwood** . 196
**Everybody's Page** . . . 197
**Notes and News** . . . 198
**Scraps and Gleanings—General Charities** . . . 199
**Annotations.**—Music and Medicine.—Why are Sixpenny Doctors Objectionable?—The Mathews Duncan Memorial 200
**The Lords' Committee** . . . 204
**The Practitioner's Mirror and Retrospect**—
MEDICINE.—Auscultatory Percussion, 201; The Pathology and Treatment of Erysipelas . . . 202
SURGERY.—Surgery of the Pericardium . . . 203
THERAPEUTICS.—Thuja Occidentalis . . . 203
NEW DRUGS, APPLIANCES, AND THINGS MEDICAL . . 203
**London Samaritan Society's Convalescent Homes** 204
**Turning Over New Leaves.**—Antiseptics in Obstetric Nursing . . . 204
**The Student of Medicine.**—Editorial Notes.—Notes from the Schools.—Appointments.—Vacancies.—Pass Lists

### EXTRA SUPPLEMENT—THE NURSING MIRROR.

**En Passant.**—Zenana Work—Short Items—Asylum Attendants—Holiday Hints—Stockton Nursing Association—The Hebrides—Sick Pay . . . xcv

**Protecting the Public** . . . xcvi

**Everybody's Opinion.**—For the Lepers . . . xcvi

**Examination Questions** . . . xcvi

**Asylum Articles.** IV.—Comparisons . . . xcvii

**For Reading to the Sick**—Sympathy . . . xcvii

**Trained Nurses Club** . . . xcviii

**Lectures on Surgical Ward Work and Nursing.** By ALEX. MILES, M.B. Edin., C.M., F.R.C.S.E.—Lecture XXXI.—Bandages for Upper Extremity . . . xcviii

**Notes from Australia** . . . xcix

**Notes and Queries** . . . xcix

**Off Duty.**—An Urgent Case . . . c

**A Holiday Amusement** . . . c

**Appointments—Christmas Competitions** . . . c

**Amusements and Relaxation** . . . c

